# Supplementary material for: Star Polycation Mediated dsRNA Improves the Efficiency of RNA Interference in Phytoseiulus persimilis
Source: Nanomaterials (Basel). 2022 Oct 28;12(21):3809. doi: 10.3390/nano12213809 (PMC9656875; doi:10.3390/nano12213809)
Supplement: Supplementary file 1 [file nanomaterials-12-03809-s001.zip › nanomaterials-1968390-supplementary.pdf]

>ATP subunit b *Phytoseiulus persimilis*
ATGCTGTCGAGAGTGGCCATTGCGAGGAAGCTCGCCCCACTCACTGTAGCCAATGTCAGGTGTT
CCAGCAGTCAACCCATCTGGAAACGCGAAATTCTCGTGGTGAAGGAGCACCCCGATAGAGATC
TGGTCAACTTTCCGAGATACAAAATGCCAGAACACCCATCGCCAGTGAGGATGGGCTTCATACC
TGAGGAATGGTTCCAGTTTCTCTATCCGAAAACAGGGCTCACGGGACCCTATGTGCTTGGCGCC
AGTCTGATCACAACCATACTCTCCAAAGAGTTCATGGTGGTCGAAGAAGAGTTCATGTTCGGCC
TCGTATTCTTCTCAGGAGTTGCCGTGCTGCACAAGTTGTACGGCAAAGACATAGCCGCTGCTATT
GATACTGTTATGGAGAAGGAAGCAGCAGAGGAGGCGAAGGGATTGAATGAGAAACGTCAATT
CATCGAAGACTCGATCAAACATGAGGAAACCGCCCAACTTCAAACCGCCGCCCAGGAGATTAT
ATTCGCAGCCAAAAAGGAGAACGTTGGCTTTCAACTGGAAGCCGAGTTCCGTAAACGACAGCT
TCAGGCATGTTAA

> ATP subunit d *Phytoseiulus persimilis*
ATGGCTCGACGCATCACGAAGTCAGCCGTCGACTGGGTGAAGTTGGCTGAGCTGGTTCGGAAA
GAGGAAATGCCGCATTTCGCCGCTTTCCGCGCTAAAAGCGACAATTACGTGCGCGTCGTTCATG
AGCTTCCCGAGGAGCTCCCCGCCATCGACTTCGCGCACTACAAGTCCCGGATCGCGAATCCCA
AACTTGCCGAGGAATTCGAGCAGAAATACAAAGCCATCAAGATCCCGTATCCAGAGGACAAGT
ACTCCGCGCAGATCGATATGATGGCCGTAGAAACGAAGAAAGAGATTGCCGCTTTCATTGCTG
GTTCCAAGAAGCGAATCGAAGAATTGAAAGCCGAACGACAGAAACTCGATGACATGATTCCCC
TTGAGCACATGACAATGGAGGACTTCGCAGAGGCATTCCCTAATGAGACCTGGAACCCGGACA
AACCAACCTTTTTCCCACACGACCACGAGACTCAGCAATTAATCAAGGAGGGAATCGAAGAGG
AAGCTCGCGGGGCCCATTGA

>*PpRpL11*_CDS *Phytoseiulus persimilis*
ATGACGAAGACTAGGGTGCCACCTTCCACGAGAGCCGCCGACAAAACTGTCGCCGGCAGAGTT
CAGAAAGACAAGGAAAAGAACTCGATGCGGGATATCAAGATCCGGAAACTCTGTCTCAACATT
TGCGTCGGAGAATCCGGAGATAGGCTGACTCGTGCTGCAAAGGTGTTGGAACAGCTCACTGGT
CAGAACCCCGTCTTCTCAAAAGCTCGTTACACTGTGCGATCCTTCGGCATCCGTCGTAATGAGA
AAATCGCCGTCCACTGCACTGTGCGTGGCGTGAAAGCTGAGGAAATCCTCGAGAAAGGTCTCA
AGGTCCGGGAATACGAACTACGCAAGGACATGTTCTCCGACACAGGCAACTTCGGCTTCGGAA
TTCAGGAACACATTGATCTGGGTATCAAGTATGATCCTTCCATCGGTATCTATGGTCTCGATTTCT
TCGTCGTTCTCGGTCGAGCTGGATACAACGTGCCTCACCGTAGGAGGAAGACCGGGAAGGTTG
GAATCCAGCACCGACTGACGAAAGAAGAGTCGATCAAATGGTTCCAGCAGAAATACGACGGA
ATCATCCTCCCTCCCAAGAAGAAGTAA

>*PpRPS2_* CDS *Phytoseiulus persimilis*
ATGTTTGCGTCAGTCCGCCGCAATATGTGGCTGAAATTCAAATGGACCGCCTCTTCAACTCTCTC
TTCTCCTGCCTGGCTGCCAAAACAGCGGGCTGAAGCGACACCGAAACCACCGCGTCGGTCCTT
TTGTTCCGAAGAAGAGTCGACAATGGCGGATGCAGCTCCTGCCGGTCGTGGCGGTTTTCGTGGA
GGTTTCGGTCGAGGTGGCCGTGGCGGACGTGGACGCGGTCGTGGTCGTGGACGTGGAGGGCG
TCGGGGAGGCAAGGAAGGCGAGAAGGAGTGGCAACCAGTGACGAAACTTGGGCGTCTCGTCA

AGGATGGGAAGCTCAAGACCCTCGAGGAGATCTATCCGTATGCCTACCCTATCAAGGAATTTGA
GATCATCGACGCTTTTCTTGGAACGACTCTCAGAGACGACGTCCTGAAGATCATGCCTGTCCAG
AAGCAGACTCGCGCCGGTCAAAGAACACGTTTCAAGGCTTTTGTGGCCATCGGTGACTACAATG
GCCACGTTGGTCTCGGTGTCAAGTGCTCCAAAGAAGTTGCCACCGCCATTCGTGGAGCCATCGT
GCTGGCGAAACTGTCTGTCATTCCAGTCAGAAGAGGATATTGGGGTAACAAGATCGGTAAACC
CCACACCGTGCCTTGTAAGGTTTCTGGAAAGTGTGGGTCCGTGAGCGTGAAGCTCATTCCCGCG
CCGCGTGGAACTGGCATCGTGTCTGCCCCCGTACCGAAGAAACTCCTGCAAATGGCTGGAATC
GATGATTGCTACACTCAGGCTCGGGGCTCGACTTGCACCCTTGGCAACTTCGCCAAAGCCACCT
ATCTGGCCATCCAGCAGACGTACTCCTATCTGACTCCGGACTTGTGGGCCGATCAGCTTTTGAG
CAAAACGCCCTATCAAGTACATACGGACCATCTTTCGAGCGCAAACAAGTCCCACGCTCATTAT
TCACATGAATAG

>*Pptra2_*CDS *Phytoseiulus persimilis*
ATGATTTCGTCGCCCACAGAGTGTGTTGTGTCGGCAGCGGCGTTGGCGGCGGCGAGCTCCTCGT
TGTTGCTAGTGTGTGTGTATTTCGCAGTGTGCTAGCGGCTGCTGTTGGTGTTGGATGAAATACGC
GATTCGTACCTCGTCGTTTCTTTGTCCTTCATATCTCCGAGAGATGTTTATGTTCGCGGAGATTCG
CACTCCCGATCGCGTTCTCGATCACGGTCTGACCGTGACAGAGAAGATCGGCGTAGCAGGAGT
CGTAGCCGAAGTAGAGATCGCGAATCAAGGCGAGACCGGAAGGACAGACGGTCCCGCAGCCG
GTCCCCCGTAGGAAGCAGGTACGATGGAGACGAAGGAAAACGTCACTCCGGCGATCGAGAAC
GCCCACATCCTTCGCGTTGCATCGGGGTTTTCGGCTTGTCGATCTACACCAACGAGCGCGAGCT
CAGAGATTTTTTTGCGAAGTACGGCCGCGTTGAAGACGTTCAGGTTGTCTACGATGCCCAGA
CTGGACGTTCGCGAGGTTTCGGATTCGTCTACTACGAATCCGAAGACGATGCTCACGAAGCCAA
GGAGAAAGCCAACGGCCTGGAAATCGATGGACGCAAAATCCGTGTCGATTTCTCGATCACCAA
GAGAGCTCACACGCCCACTCCCGGAGTCTACATGGGTCGCCCCACAAGAAGACCGAAGCGAG
GATCAGGAGGCGGAGGTGGTGGTTATGACCGAGGAGGCAGCGATCGGGGCAGCCGAAGCCG
GAGATATACTCGATCGCCTTCACCGTACCGAGAGAGACGCGGCGGTGGCGGAGGTGGCAGGG
ACCGTTATCGTGAGCGCGAGAGATCGTACTCGCCTCGGAGATACTATTAG
